# B-type natriuretic peptide and N-terminal Pro-B-type natriuretic peptide in severe aortic stenosis: a comprehensive literature review

**DOI:** 10.3389/fcvm.2023.1182530

**Published:** 2023-09-01

**Authors:** Pâmela Nogueira Cavalcante, Gabriel Kanhouche, Vitor Emer Egypto Rosa, Carlos M. Campos, Mariana Pezzute Lopes, Maria Antonieta Albanez A. de M. Lopes, Roney Orismar Sampaio, Fábio Sândoli de Brito Júnior, Flavio Tarasoutchi, Alexandre Antonio Cunha Abizaid

**Affiliations:** ^1^ Instituto do Coracao (InCor), Hospital das Clínicas HCFMUSP, Faculdade de Medicina, Universidade de Sao Paulo, Sao Paulo, Brazil; ^2^ Departament of Hemodynamic, Instituto Prevent Senior, Sao Paulo, Brazil

**Keywords:** biomarkers, natriuretic peptides, B-type natriuretic peptide, aortic stenosis, aortic valve replacement

## Abstract

B-type natriuretic peptide (BNP) and N-terminal pro-BNP (NT-pro BNP) are cardiac biomarkers that are released in response to increased ventricular and atrial wall stress. Aortic stenosis (AS) leads to hemodynamic changes and left ventricular hypertrophy and may be associated with natriuretic peptide levels. Several studies have shown that increased natriuretic peptide levels are correlated with AS severity and can predict the need for intervention. It can be useful in risk stratification, monitoring follow-up, and predicting cardiovascular outcomes of patients with severe AS. This paper aims to summarize the evidence of the role of BNP and NT-pro BNP in AS, before and after intervention.

## Introduction

Biomarkers are measurable laboratory indicators that could be helpful in the diagnosis or in the therapeutic decision of certain diseases. Several biomarkers have been studied in aortic stenosis, but B-type natriuretic peptide (BNP) is currently the only biomarker that is supported by valvular heart disease guidelines recommendations (1). BNP is a cardiac hormone mediating natriuresis, diuresis, and vasodilation (2). It is released in response to increased ventricular and atrial wall stress (1–3). Elevated BNP levels may reflect ventricular hypertrophy and cardiac dysfunction (3). BNP is cleaved from its prohormone, pro-BNP, and the N-terminal fragment, N-terminal pro-BNP (NT-pro BNP), is also released into circulation. NT-pro BNP has a similar diagnosis and prognostic implication to BNP, however, the evidence is weaker in the context of aortic stenosis (AS) (4).

AS is the most prevalent valve heart disease in the general population aged over 65 years, mainly in developed countries (5). The pathological process that occurs in aortic stenosis leads to hemodynamic changes and left ventricular hypertrophy (2). During the systole, aortic stenosis increases the resistance to the work of the left ventricle (LV). As a result, LV adapts to increased afterload by myocardial remodeling, which allows for the restoration of wall tension. This mechanism is a physiological response to normalize wall stress and, thus, maintain contractile function (1, 2, 6). However, in the natural evolution, LV may decompensate, and cardiac output decreases (6). Ventricular hypertrophy is correlated to BNP levels (3). Chen et al. (7) suggested that the hypertrophic response and BNP expression were controlled by a common pathway, but the exact mechanisms behind the release of BNP and how they lead to LV remodeling are still uncertain (1, 6, 7).

The role of natriuretic peptides as cardiac biomarkers in congestive heart failure (HF), as well as the prognostic value in patients with HF, is well recognized (8). BNP levels are correlated to New York Heart Association (NYHA) symptom class and are predictors of cardiovascular outcomes in HF (8, 9). Additionally, they are useful for discerning between cardiac and non-cardiac dyspnea, except for patients with AS (10). Previous studies showed that BNP levels correlated with severe AS and its echocardiographic parameters of severity (11–13). Furthermore, the onset of symptoms may be correlated with enhanced BNP levels (12). The use of BNP as a method of risk stratification of patients with AS is also a great research interest area, especially in the timing of intervention (14).

Despite this, the relative importance of BNP and its utility as a cardiac biomarker in aortic stenosis is currently under discussion. This paper aims to summarize the evidence of the role of BNP and NT pro-BNP in aortic stenosis, before and after interventions, highlighting a comprehensive analysis of their diagnostic and prognostic significance, which thus sets this paper apart from other reviews in the field. Boolean searches on PubMed, Medline, and Google Scholar included terms related to BNP or NT-pro BNP with terms for aortic stenosis and aortic valve replacement. The data analysis focused on randomized controlled trials, meta-analyses, and cohort studies conducted between 1995 and 2023. A total of 60 papers were reviewed excluding review articles and others less relevant to the topic.

### Natriuretic peptides and severe aortic stenosis

The first studies published correlating BNP levels to severe AS demonstrated that increased natriuretic peptide levels are correlated with the mean pressure gradient, peak velocity, peak transaortic gradient pressure, aortic valve area, and functional status (NYHA symptom class) (11–13). Qi et al. (11) described that higher natriuretic peptide values may result not only from LV hypertrophy but also increased left atrial pressure caused by LV diastolic dysfunction. A stronger association was found between natriuretic peptides and LV mass index, aortic valvular area index, and mean aortic valve gradient. After multivariate analysis, only BNP remained as a predictor of these echocardiographic parameters (11).

In 2004, Weber et al. (12) found a positive correlation of NT-pro BNP levels to the mean transaortic pressure gradient, linked to the severity of AS and NYHA class, and that NT-pro BNP levels can serve as a recommendation for aortic valve replacement (AVR). AUC for NT-pro BNP to discriminate the need for intervention was 0.73. Their results were in agreement with the data published previously (12, 15, 16). Most of these studies excluded patients with reduced LV ejection fraction and valve disease concomitant with AS, which might cause elevated natriuretic peptide levels (15, 16).

Other studies have identified a progressive increase in BNP and NT-pro BNP values with decreasing aortic valve area but included a large increase in patients with LVEF <50%, which may emphasize the association between natriuretic peptide levels and cardiac function (11, 17).

These findings suggest that BNP is related to AS severity and could be useful to monitor asymptomatic patients (14).

### Natriuretic peptides and aortic stenosis symptoms onset

The onset of symptoms is a critical point in the natural history of AS and is a hallmark for the recommendation of AVR. In some patients, the development of symptoms related to severe AS, such as exertional dyspnea, is unclear, non-specific, and often difficult to be evaluated clinically (12).

The use of BNP as a biomarker reflecting early decompensation in asymptomatic patients had been demonstrated previously in several studies (12–16). Gerber et al. (12) identified a strong association between NYHA class and natriuretic peptide plasma levels. It was higher in patients with NYHA class II than in those with class I symptoms, emphasizing that BNP could be used to distinguish between early-onset symptoms and normal effort tolerance. In contrast, they did not find a correlation between syncope or angina with enhanced natriuretic peptide levels, which suggests that these symptoms may have different pathophysiology in AS (12). These findings were corroborated by Lim et al. (18), who observed that BNP serum level >66 pg/ml detected symptomatic patients with increased NYHA functional class with a sensitivity, specificity, and accuracy of 84%, 82%, and 84%, respectively (18).

The optimal timing of intervention in asymptomatic patients with severe AS remains a challenge in clinical decision-making, and monitoring BNP levels may be helpful in this context. However, measurements of BNP do not substitute clinical and echocardiographic evaluation of these patients.

### Natriuretic peptides in aortic stenosis grading classification

In asymptomatic patients, the relationship between classical parameters of AS severity, in cases of normal flow and high gradient, with elevated natriuretic peptide levels and poor prognosis is well established. In contrast, the association of biomarkers and prognosis for other AS categories remains unexplored. Results from a cohort study with specific subgroups of AS showed that patients with normal flow and low gradient who had lower BNP levels exhibited the best prognosis. While patients with true low flow and low gradient who had higher BNP levels displayed the worse outcome, with more LV remodeling and increased global LV afterload, similar to those patients with normal flow and high gradient (19). Results from the TOPAS study corroborated these findings, demonstrating that, in patients with classical low flow-low gradient AS (CLFLG-AS), BNP and NT-proBNP were powerful independent predictors of all-cause mortality. Furthermore, high levels of natriuretic peptides could accurately identify high-risk patients that could have survival benefits from early intervention. In addition, it was the first study to reveal that the prognostic value from NT-proBNP appears superior to that of BNP for this specific group of CLFLG-AS patients (20).

Moreover, the prognostic value of natriuretic peptides combined with high-sensitivity troponin T (hs-TnT) could be enhanced in LFLG-AS patients who had either preserved or reduced LV ejection fraction. Elevations of both BNP and hs-TnT were associated with a significantly higher risk of cardiovascular mortality in this group (21). Furthermore, higher levels of BNP and hs-TNI combined were also associated with progressive worsening of imaging parameters of LV remodeling and fibrosis by cardiac magnetic resonance (CMR) and poor outcomes in CLFLG-AS patients (22).

### Natriuretic peptides and subclinical left ventricular dysfunction in aortic stenosis

Studies correlating natriuretic peptides and biomarkers, such as troponin or imaging modalities, e.g., LV global longitudinal strain (LV-GLS), can provide synergistic risk stratification, independent of symptoms, risk factors, and other severe echo parameters in critical patients with AS. After aortic valve replacement, reversal of LV remodeling with regression of myocardial hypertrophy was observed, leading to an improvement in LV-GLS and decreased NT-pro BNP levels. However, more prospective studies are needed before natriuretic peptides can be used routinely to determine valve intervention timing (23, 24).

### Natriuretic peptides and moderate mixed aortic valve disease

Mixed aortic valve disease (MAVD) is defined as an association between AS and aortic regurgitation (AR). In patients with moderate-to-severe MAVD, high BNP levels were correlated with worse LV-GLS, higher LV-mass index, elevated left atrial volume index, and higher prevalence of moderate or greater tricuspid regurgitation, which are significantly associated with critical clinical outcomes. Previous studies demonstrated worse outcomes in patients with moderate MAVD when compared to those with either moderate AS or AR alone (25).

### Natriuretic peptides and cardiovascular outcomes in aortic stenosis

The prognostic value of natriuretic peptides was demonstrated by several authors (26–31). Bergler-Klein et al. (26) showed that preoperative NT pro-BNP independently predicted the risk of death and postoperative outcomes. Symptom free-survival at the 12-month event rate was 31% vs. 92%, with NT pro-BNP <80 pmol/L vs. ≥80 pmol/L, respectively (*p* < 0.001) (26). In addition, Lim et al. (18) identified that in both symptomatic and asymptomatic patients, higher BNP was a strong independent predictor for cardiovascular death and may be helpful for risk stratification in AS (18).

Pedrazzini et al. (31) showed a comparison between logistic EuroSCORE and BNP in predicting in-hospital and late postoperative mortality in patients with severe AS undergoing AVR. BNP, but not logistic EuroSCORE, was shown to be an independent predictor of mortality. BNP levels >312 pg/ml could discriminate survivors from non-survivors with a C statistic of 0.80 and 0.75 for the perioperative and overall mortality, respectively (31).

The relationship between AS and sudden death is well-recognized (16). Previous studies suggested that adaptative LV hypertrophy leads to the development of myocardial fibrosis and it may be associated with an increased risk of cardiac events and mortality in AS (32, 33). Furthermore, BNP levels are associated with LV hypertrophy, which could explain the correlation between higher BNP and cardiac mortality (33).

The plasma level of pre-procedure BNP has also been linked with the left ventricle mass index after surgical AVR in early and late evaluation, and this is closely related to unfavorable outcomes and worsening of symptoms in the post-procedure follow-up (34). The study by Iwahashi et al. (34) showed that elevated BNP levels preoperatively were associated with NYHA >I in the late evaluation of post-surgical AVR follow-up, and BNP> 312 pg/ml had a lower event-free survival rate compared to patients who had BNP ≤ 312 pg/ml (34).

Importantly, Clavel et al. (16) showed in a prospective observational study a prognostic value for the BNP activation, defined as the ratio between the measured BNP, and maximal normal BNP value specific to age and sex in patients with severe AS. Elevated BNP clinical activation was associated with higher mortality, independently of baseline characteristics, emphasizing the role of BNP as a strong predictor of mortality. The level of BNP activation was also correlated to mortality in asymptomatic patients, with an adjusted HR of 7.38 when the BNP ratio ≥ 3 (16).

Thus, ESC/EACTS and AHA/ACC guidelines recommend intervention in patients with asymptomatic AS and BNP levels >3 ×  normal, as a class IIB recommendation (16, 18, 26, 35–37).

### Natriuretic peptides in transcatheter aortic valve replacement

Recent evidence in studies on transcatheter AVR (TAVR) showed superiority in primary outcomes compared to conventional surgical AVR in most scenarios (38). In fact, the technical evolutions of TAVR operators and devices favor the improvement of results with this strategy, thus becoming a very reproducible possibility for the treatment of elderly patients with aortic stenosis, including intermediate and low-risk patients (38).

Natriuretic peptide values were also evaluated in patients submitted to TAVR and were related to prognosis (39, 40). High levels of pre-procedure BNP negatively influenced short and midterm outcomes. In addition, high BNP levels were associated with atrial fibrillation, kidney injury, lower ejection fraction, higher transaortic gradient, and smaller valve area (40). Nevertheless, high levels of BNP were found to be an independent factor for overall mortality (39). A sub-analysis of the PARTNER 2 study showed that there is a J-association of BNP and all-cause mortality in 2 years (7). Both very low and high values of this marker were associated with increased mortality. Ventricular hypertrophy in response to AS was associated with increased mortality, and elevated BNP levels were correlated with the degree of hypertrophy (7, 40). On the other hand, low BNP levels represent an inadequate ventricular response to elevated afterload of AS. The absence of hypertrophy increases LV wall stress, leading to irreversible damage to the left ventricle, such as stiffening and fibrosis, and thus increasing mortality. The patients with low BNP also presented increased troponin, which may infer myocardial injury (40). Thus, earlier intervention in aortic stenosis in this profile of patients could possibly avoid irreversible myocardial damage (7). Irreversible myocardial remodeling was observed in patients with elevated basal BNP who maintained elevated BNP levels at the 2-year follow-up. In general, after the procedure, BNP levels rise, except in patients whose levels are already high, and decrease in the majority of patients in 6- to 12-month follow-up (40). Approximately 40% of patients will persist with elevated natriuretic peptides despite TAVR, suggesting that other factors, beside the high afterload pressure, may be involved (41).

Post-TAVR BNP value also has clinical and prognostic correlation. BNP values after discharge were analyzed in patients included in the OCEAN-TAVI multicenter registry in order to evaluate prognosis and correlations with the patient profile and procedure (42). The cutoff point for increased mortality at 2 years was BNP >202 pg/ml. This profile of patients had more comorbidities, with higher age, lower mean LVEF, higher surgical risk scores, and a smaller effective aortic orifice area. In addition to mortality, more hospitalization for heart failure was also observed in patients with high BNP levels (43). Furthermore, a Portuguese study showed that 1-month post-procedure NT-pro BNP higher than 2,500 pg/ml was an independent risk factor for 1-year mortality. On the other hand, baseline NT-pro BNP did not predict outcomes (44). Previously, the prognostic value of NT-pro BNP was demonstrated by Lopes-Otero et al., who emphasized NT pro-BNP as a predictor of short and long-term outcomes after TAVR (45).

**Figure 1:**
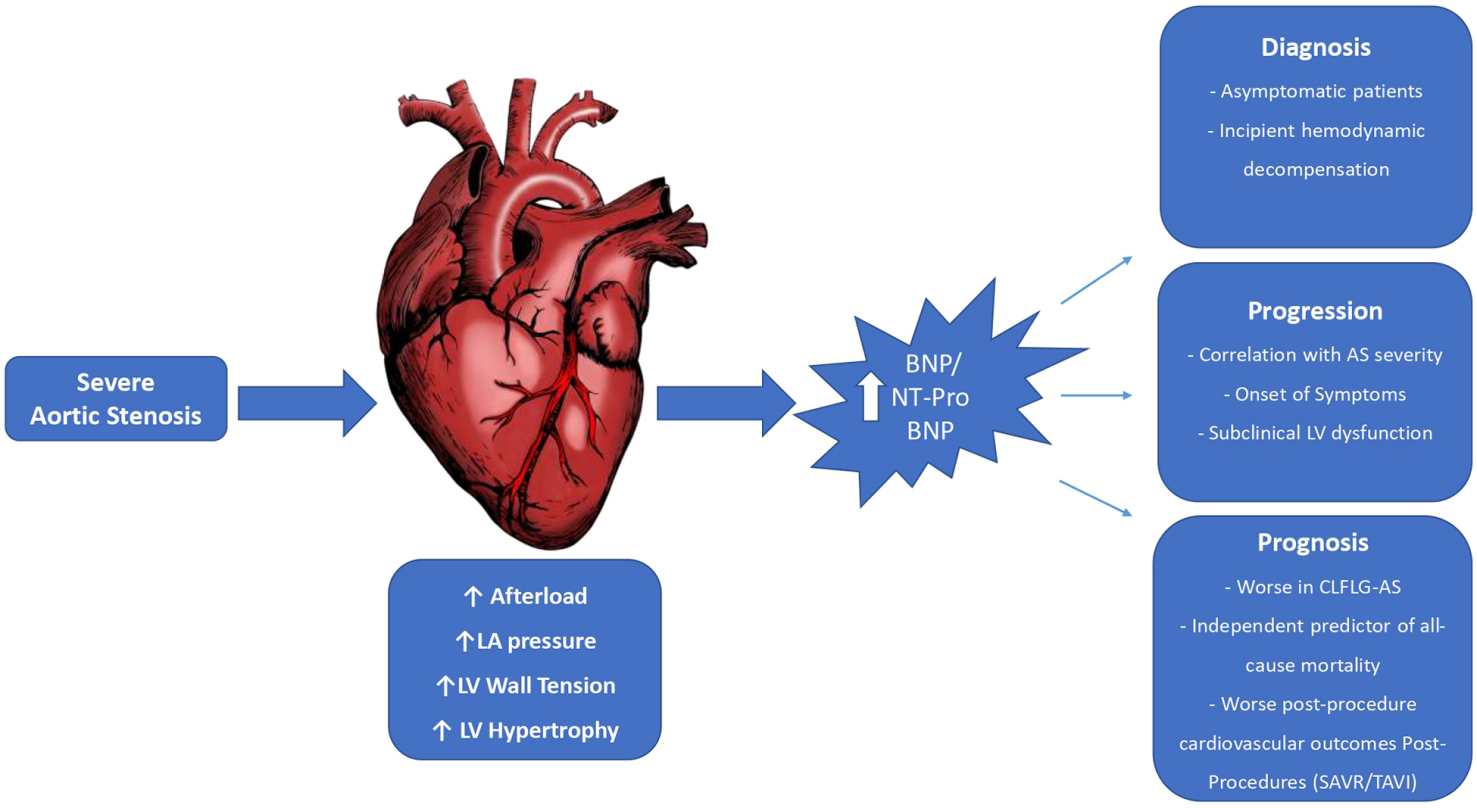
B-type natriuretic peptide and N-terminal Pro B-type natriuretic peptide in severe aortic stenosis. AS, aortic stenosis; BNP, B-type natriuretic peptide; CLFLG-AS, classical low-flow low-gradient aortic stenosis; LA, left atrium; LV, left ventricular; NT-ProBNP, N-terminal pro-B-type natriuretic peptide; SAVR, surgical aortic valve replacement; TAVR, transcatheter aortic valve replacement.

Figure 1 summarizes the major utilities and main correlations of natriuretic peptides (BNP and NT-proBNP) with different clinical aspects of AS.

### Limitations of natriuretics peptides

Various factors can lead to misinterpretation, either overestimating or underestimating BNP value. For instance, renal dysfunction, advanced age, female sex, atrial fibrillation, inflammation, hyperthyroidism, and the use of sacubitril/valsartan can cause an overestimation of BNP levels. On the other hand, certain factors including obesity, the immediate period following acute coronary syndrome onset, and the presence of pericardial effusion can result in an underestimation of BNP levels (46). Thus, in patients with AS associated with such conditions, we must assess natriuretic peptides with caution.

## Conclusion

BNP is a prognostic marker in asymptomatic patients with severe AS, and values above the 3 ×  normal level may help indicate intervention (16, 18, 26, 35–37). BNP and NT-pro BNP also show promise for detecting symptoms onset and AS severity, but further studies are needed to validate their use in this context. Post-procedure BNP also appears to be a predictor of worse outcomes. BNP and NT-proBNP combined with other biomarkers or imaging modalities could enhance diagnostic accuracy and provide a comprehensive assessment of cardiac function and structure, especially in asymptomatic AS patients, thus guiding therapeutic management. However, more prospective studies are necessary to determine a cutoff value and whether changes in the levels of natriuretic peptides could be used appropriately to decide the best valve intervention timing.
